# Paralysis Which Attends Dentition

**Published:** 1850-07

**Authors:** 


### ARTICLE III.

### *Paralysis which attends Dentition.*

Dr. Fliess, of Neusalz, has published, in the *Journal für Kinderkrankheiten*, for July and August, 1849, the following memoir on the paralysis which sometimes occurs in children during dentition.

The extraordinary progress which has been made in neuro-pathology, by the labors of English, French, and German Physiologists, has only lately caused especial attention to be directed to the paralytic affections with which young children are attacked. These affections are not unfrequent, but have been long known. By some, they have been mistaken for rheumatism, even though the child complained of no pain; while others have treated them as the effects of a blow or contusion. And we meet with cases in which sudden paralysis of a limb has been even considered as a sign of rudeness or naughtiness, just as the nocturnal enuresis of children was until a short time ago; for which children used almost always to be chastised with rods, or in some other way, and of which we have only lately become convinced that it depends on a paralysis of the muscles which close the bladder, or rather on an excessive excito-motor activity of those which expel the urine.

In using here the term Paralysis of Dentition (*Dentalparalysen)*, we would have no other paralysis to be understood than that which arises from dentition. We intend first to describe the mode of attack of these paralytic affections, and then to endeavor to point out their cause.

Paralysis is much less frequent during the first, than during the second dentition; at least, we have not had an opportunity of observing its occurrence in the first dentition. It is possible that, as children at this time have not yet the full use of their limbs, the affection may more readily escape notice than during the second dentition, when the children can run about, and employ their limbs in performing various actions. We believe it, however, to be well established, that convulsions are much more frequent than paralysis during the first dentition; and that, in the second, the ratio is changed, or at least, paralysis is as frequent as convulsions. We take this opportunity of remarking that the older the individual is, the less frequently do convulsions supervene on dentition; while paralytic affections, and, at a later period, especially during the development of the posterior molars, neuralgia, are much more frequent. We shall hereafter have occasion to make use of these facts.

The attacks of paralysis of dentition are almost always sudden. A child is cheerful, takes exercise, and plays as usual, has a good appetite, and goes to bed in the evening apparently quite vigorous. At first, perhaps, it sleeps very quietly; but it soon throws itself about in a restless manner, groans and screams out in its sleep, grinds with its teeth, is thirsty, has some heat of head, and is rather feverish towards morning. On the next day, when perfectly awake, it is unable to use one of its arms, or, in rare cases, an arm and a leg. If the arm alone be paralyzed, it hangs down; it is indeed warm, but, in consequence of the gravitation of the blood in the limbs, the joints of the hand and the fingers are blue-red and swollen. The child cannot move the arm; sensibility is either entirely lost, or is very obtuse; and the excito-motory power can but little, if at all, be excited by strong stimulation. If a medical man be summoned, who does not think of paralysis from dentition, or knows nothing about it, he does not know how to reconcile the sudden attack of paralysis with the otherwise apparently healthy state of the child, which, as has been said, has but little fever, and complains of no pain whatever. He gives it as his opinion that the child has probably laid on the arm during the night, and pressed on the nerve; and he leads the parents to hope that, in a day or two, the effect will spontaneously be removed, just as, in a limb benumbed or asleep through pressure, the removal of the pressure is followed by a return of its power. But when, after some days, this result does not follow, but the limb remains paralyzed, and the parents become more anxious, the medical attendant is puzzled; he suspects that something of more importance is at the bottom, and he prescribes accordingly. But what does he order? Friction with opodeldoc, camphorated spirit,

&c.; and, then, perhaps, imagining that there is some deposit, he goes on to order friction with iodine ointment, mercurial ointment, embrocations, &c. We have seen a case in which the rotary electro-magnetic apparatus was diligently used; nevertheless, the arm remained paralytic, and at last became atrophied. In another case, the medical attendant could not be divested of the idea that there was a subluxation: he pulled the child's arm about, until, having had his attention called, by a colleague, to the utter loss of power in the limb, and to the great impairment of sensation, he was convinced of the existence of paralysis.

The paralyzed arm usually hangs down as if dead; and, if the child at last complain of pain, it is from dragging of the muscles of the shoulder. The paralysis sometimes extends to the leg of the same side; the child is then unable to move it, and the sensation is lost, or at least impaired. More rarely, one arm and both legs, or both arms, without the legs, are affected.

The duration of the paralysis is very uncertain; in many cases, it lasts from a fortnight to three weeks, in others many months, and even, in some, many years, and then becomes incurable. Convulsions not unfrequently precede or occasionally accompany the paralysis, without any cause, or from a very slight one. If the child be in a passion or annoyed about any thing, the paralyzed limb sometimes becomes convulsed, and performs various movements which the child has not power to restrain. These convulsive movements are also produced by the use of electric shocks; or evident vibrations of the muscles manifest themselves, on the application of a continued electric current by means of the rotary apparatus. The cases which we have observed were not preceded by convulsions; but several colleagues have observed their occasional occurrence. The termination is not unfrequently favorable, under judicious treatment; the paralysis passes off gradually, and at last entirely ceases, with a pricking or stinging sensation, or a feeling of formication. But not unfrequently the paralysis is obstinate; it resists the ordinary remedies; and other symptoms, indicating an affection of the spinal cord and brain, appear. The child is attacked with dyspnœa, palpitation, twitchings of the muscles of the eyes; it begins to squint, becomes dull, falls into a comatose state, and dies. The paralysis may become chronic; there are then no obvious indications of affection of the spinal cord or brain, but the affected limb remains paralyzed, and ultimately becomes atrophied; the individual may be, in other respects, in the most flourishing health, even active and strong. Cases have been sometimes met with, in which individuals of muscular and powerful build have had an arm paralytic and arrested in development; and, when asked as to the origin of the paralysis, they can give no other answer than that they have had it from childhood, and that a cold, negligent treatment by a physician, a fall, a blow, or whatever they think must be assigned, must have been the cause.

There, are, unfortunately, but few autopsis recorded, which can give an explanation of this form of paralysis. This perhaps depends on the circumstance that the affection is seldom fatal; at most, the opportunity is afforded in chronic affections of this kind, when death has resulted from the supervention of some disease, or from an affection of the spinal cord or brain.

But then the appearances presented are rather to be considered as results of the disease, which has more lately supervened; at least, it is then difficult to distinguish the primary from the secondary lesions in the brain or spinal cord.

In one case only, we have had an opportunity of making a post-mortem examination of a boy, who, while suffering from recent paralysis of one arm, was killed by being thrown from a carriage. This is also the case which first drew our attention to the form of paralysis under consideration.

Case.—Rudolph Meyer, the son of a tobacconist of this place, was, at birth, a strong well-made child. He was nursed by his mother, who was perfectly healthy; and, at the end of the tenth month, he was weaned. The first dentition was easy, being attended only with slight indisposition, which made him rather lean. He soon recovered, and was apparently healthy up to five years of age. During this time he had hooping-cough, which went through its usual course, and somewhat weakened him; but when it was ended, his health was perfectly re-established. At the end of his fifth year, he was frequently troubled with loaded tongue, some fever, heat of head, and flushed cheeks; these symptoms soon yielded to the employment of mild purgatives. From time to time he had, for a week at a time, restless nights; he cried out in his sleep, ground his teeth, got up as if to walk in his sleep, and was, although apparently well, very tired in the morning. One morning, on awaking, after such a restless night, he had complete paralysis of the left arm. This arm hung loosely by his side; the hand was very red, and the joints somewhat swollen. The child could not perform any movement with the limb; if it were raised, it was as heavy as the arm of a dead person, and fell again as soon as it was let alone. The warmth of this arm did not differ from that of the other. It felt a little colder; but the thermometer indicated no difference of temperature. The pulse in the left arm was somewhat weaker than in the right; but there was no difference in frequency. The boy did not feel when the arm was pinched, or pricked with a needle. This loss of sensation was more obvious on the extensor than on the flexor surface of the arm; it was least on the radial side. Here, as far as could be gathered from his account, the boy had an obscure perception of the above-named irritants; and, when he slept, the arm was slightly convulsed, when irritated on the radial side. In other respects, the boy complained of no pain; he had some cough, was cheerful, played about, and scarcely troubled himself about his arm. A great variety of remedies were employed. At first, the cause of the paralysis could not be understood; an examination of the arm, shoulder, and spinal column shewed nothing. An examination of the mouth made it probable that the paralysis was connected with dentition: the anterior milk molars were found lying half decayed in the gums, and near between them were the edges of the permanent molars, in a row in the gums. We resolved to have all the remains of the teeth extracted the next day by a dentist, so as to make room for the new teeth. But on the same day, the boy met with an accident which deprived him of life. Having been taken by his parents to see a relation in the country, he was thrown on his head from the carriage, and died towards the evening.

A careful *post-mortem* examination was made. There was a considerable fracture of the skull, and also injury of the brain, which was the immediate cause of death. But the most interesting point was the state of the upper part of the spinal cord. This showed, in the vicinity of the roots of the brachial nerves, a very remarkable degree of vascularity: the membranes were here reddened, and the whole circumference seemed congested; the veins at least were here fuller, and more prominent on the left side than on the right. There was no true organic alteration to be observed, either in the spinal cord, or in the roots of the nerves, or in the brachial nerves, so far as they could be followed. On tracing the blood-vessels, it was found that the turgescence of the veins on the left side extended on the shoulder and neck, as far as the face, where all the veins were fuller and more prominent than on the right side. The veins in the neck and submaxillary regions were remarkably prominent. There appeared to be no doubt that dentition had produced this state of the veins; and, moreover, that this congestion, having extended to the spinal cord, had probably produced pressure on the roots of the brachial nerves, and thus induced paralysis of the arm.

This autopsy fully confirms Dr. Marshall Hall's views of the origin of paralysis; and, although we have no further foundation than this single case, we may here lay down certain rules for treatment, leaving it to subsequent researches to determine whether they are perfectly correct. The rules, which we deduce from our observations, are the following:

1. When a child, during the first or second detition, is suddenly seized with paralysis of one arm, or of the whole side of the body, or is affected in only a part of the same, without any obvious external cause, this is to be considered as a *paralysis from dentition.*

2. An accurate examination of the mouth will confirm the diagnosis; for the teeth will be found firmly compressed within the gums. Generally, the molar teeth are at fault; much more seldom the incisors; at least in those cases, which we have found recorded by various writers, where the situation of the teeth has been given, the molars have almost always been mentioned as in progress of escaping from the gums.

3. The consequence of this dental irritation is obviously an increased sanguineous congestion. In some children, this may extend to the brain, and thus produce convulsions or some cerebral affection; in others, however, the congestion is limited, for some unknown reason, to the external veins, and extends to those which are between the muscles, and even to the vertebral veins and their minutest branches, so that the roots of the brachial nerves are compressed. Hence arises paralysis, which is generally confined to that side where the dental irritation has given rise to congestion.

4. By obviating the venous plethora, the pressure on the roots of the nerves, and consequently the paralysis, are prevented. But, if the congestion be intense and permanent, the pressure may produce partial atrophy of the roots of the nerves, and consequent permanent and incurable paralysis.

5. The application of stimulants to the paralyzed limbs, or even the use of electricity, can produce no effect. Repeated cupping in the neighborhood of the origins of the nerves, scarification of the gums, wrapping the paralyzed limb in flannel, mild purgatives, can alone produce any result.—*Lond. Jour. Med.*—*Am. Journ. Med. Sci.*

